# Editorial: Novel insights into the pathophysiology of diabetes-related complications: Implications for improved therapeutic strategies

**DOI:** 10.3389/fendo.2023.1157807

**Published:** 2023-03-01

**Authors:** Jian Ma, Chunjie Jiang, Xuebin Fu, Jun Chen, Wenxiang Hu, Lin Yuan

**Affiliations:** ^1^ Department of Immunology, Harbin Medical University, Harbin, China; ^2^ Department of Genome, University of Texas MD Anderson Cancer Center, Houston, TX, United States; ^3^ Department of Cardiovascular-Thoracic Surgery, Northwestern University Feinberg School of Medicine, Chicago, IL, United States; ^4^ Department of Pediatrics, Ann & Robert H. Lurie Children’s Hospital, Chicago, IL, United States; ^5^ Department of Endocrinology, Qilu Hospital, Cheeloo College of Medicine, Shandong University, Jinan, China; ^6^ Department of Basic Research, Guangzhou Laboratory, Guangzhou, China; ^7^ Laboratory of Research in Parkinson’s Disease and Related Disorders, Health Science Institute, China Medical University, Shenyang, China

**Keywords:** diabetes, complications, pathophysiology, molecular mechanism, insight

Globally, diabetes mellitus (DM) is a significant and prevalent health problem. It is increasingly apparent that not only a cure for the current worldwide diabetes epidemic is required, but also a cure for its major complications, including heart disease, chronic kidney disease, and nerve damage (1). In addition, other problems with feet, oral health, vision, hearing, reproduction, and mental health also need to be explored (2–6). Understanding the underlying mechanisms of these diabetic complications would help prevent or delay the occurrence of complications and to improve the overall health condition of people with DM. To promote the understanding, we were invited by the journal editorial team to organize A Research Topic titled “*Novel Insights into the Pathophysiology of Diabetes-related Complications: Implications for Improved Therapeutic Strategies*”. This topic aims to recruit high-quality original research and review articles that contribute to uncovering the intracellular signaling pathways with the development of diabetic complications, or exploring the possible role of genetic issues, metabolic regulation, and inflammation mechanisms. The topic was initiated last spring, May 5th, and closed on Sept 22nd, 2022. During these five months, total 37 submissions, including 25 manuscripts and 12 abstracts, were received. And finally, 10 peer-reviewed articles with high quality were selected and published. These 10 articles cover different issues on diabetes and complications, i.e., diabetic retinopathy (DR), diabetic neuropathy (DN), type 2 diabetes mellitus (T2DM)-associated periodontitis, diabetic oxidative liver damage, diabetic-related wound healing, etc.

To DR, a common and serious microvascular complication of DM, Li et al. performed a study on investigating the correlation between methylation of *THBS1* transcription regulation area and DR occurred. Based on the detection data from recruited patients diagnosed with DR and DM patients without retinal problems, they observed that THBS1 mRNA expression in peripheral blood was significantly higher in DR patients than in DM patients and concluded that *THBS1* overexpression is related to *THBS1* transcription regulation area hypomethylation and may be genetically controlled in DR patients. To another microvascular complication of DM, DN, one research article delineated the underlying mechanisms of dapagliflozin as a potent drug for DN. In this study, Bai et al., revealed that many lncRNA and mRNA expression levels were significantly altered in kidney tissues of dapagliflozin-treated *db/db* mice by combing transcriptome analysis and a network pharmacology approach. Network pharmacology analysis further identified that several genes including SMAD9, PPARG, CD36, and CYP4A12A might be the pivotal targets of dapagliflozin for treating diabetic nephropathy. Taken together, this study provides novel insights into the protective mechanism of dapagliflozin for treating nephropathy. Also, for DN, a study from Cao et al., revealed the role of S-palmitoylation in DN. In the research, the authors analyzed the proteomic data of lumbar dorsal root ganglia (DRG) of diabetic mice and palmitoylation profiling data of the HUVEC cell line, demonstrating that the S-palmitoylation status of Cys47 could affect the interaction between PRDX6 and the C-terminal domain of AE3, thereby regulating the activity of AE3 anion exchanger enzyme in the nervous system, revealing a potential association between activating protein palmitoylation and DN.

In addition, we have one article focused on T2DM-associated periodontitis, a common disease with high prevalence featuring persistent infection and complicated manifestations. Wang et al. used the rat model to mimic T2DM-associated periodontitis, which was found to have an intense inflammatory response and decreased autophagy. In the treatment group, the application of calcitriol, a common supplement, had shown a significant inhibition effect of inflammation and increased autophagy. The study provided a piece of evidence that an easy and daily supplement of calcitriol may provide effective treatment against T2DM-associated periodontitis.

For diabetic-related wound healing issue, Mu et al. focused on the role of pyroptosis in promoting diabetic complications and summarized the underlying mechanisms of pyroptosis in the recovery of diabetic wounds, such as the activation of the NLRP3 inflammasome and other pyroptosis-related signaling pathways, and highlights the potential therapeutic approaches to promoting diabetic-related wound healing *via* targeting these signaling pathways. Besides, a research article from Li et al. comprehensively analyzed differential expression genes in diabetes and depression based on several RNA sequencing datasets from the GEO database and found an interesting target molecule NK1R in the overlapping set. They further designed *in vitro* and *in vivo* experiments to confirm the function of NK1R in angiogenesis, epithelial-mesenchymal transition (EMT), collagen deposition, and inflammation in diabetes and depression. Of note, the *in vivo* experiment data suggested that the downregulation of NK1R promoted vascular proliferation and enhance diabetic wound healing, providing a potential therapeutic target for the management of diabetic non-healing wounds and depression.

Given that the disruption of circadian rhythm is associated with the development of T2DM, Jiang et al., did a comprehensive bioinformatics analysis about the rhythmicity of gene transcription, mature RNA abundance, protein abundance, and DBP activity, presenting a global view of the oscillating genes in multiple layers and indicating the complexity of regulatory mechanisms across different layers for further functional study. In addition, m6A has been demonstrated to be crucial to the development of DM and its complications. To plot the knowledge maps and predict the hotspots and trends in m6A related studies on DM, Zhang et al., used the CiteSpace software to perform a retrospective bibliometric analysis and science mapping. They provided an overview of the trend of publication outputs, countries/regions and institutions distribution, authors, cited authors, cited journals, as well as hotspots and frontiers, which may help us better understand the studies in the field. And most interestingly, one study of current topic show different result from previous research. It’s well known that long-term treatment with omega-3 PUFAs, which are CYP2E1 substrates, may affect CYP2E1 expression in the liver, and result in the development of diabetic oxidative liver damage. However, Maksymchuk et al., observed that long-term treatment of diabetic rats with omega-3 PUFAs does not increase the risk of CYP2E1-dependent oxidative stress and development of liver pathology but prevents some diabetic ultrastructural damage to hepatocytes *via* using streptozotocin-induced rat model of type 1 diabetes.

In a word, we believed that the above review or research articles could reflect the research progresses on diabetes and complications and contribute to the academic community. With a limited number of submissions in current topic, we are looking forward to seeing many more academic articles on diabetic complications studies in the future, particulars at other research targets, such as DM-related heart disease, a reproductive disorder in diabetic women (7, 8) or the relationship between diabetes and the development of Alzheimer’s disease (9), etc.

## Author contributions

All authors listed have made a substantial, direct, and intellectual contribution to the work and approved it for publication.
